# Supplementary material for: MicroRNA Biomarkers as Promising Tools for Early Colorectal Cancer Screening—A Comprehensive Review
Source: Int J Mol Sci. 2023 Jul 3;24(13):11023. doi: 10.3390/ijms241311023 (PMC10341417; doi:10.3390/ijms241311023)
Supplement: Supplementary file 1 [file ijms-24-11023-s001.zip › ijms-2469340-supplementary.pdf]

Table S1. Performance of all microRNAs evaluated the literature search in the context CRC screening both in stool and blood samples.

MicroRNA	Author, Year	N	Country	Compared Groups	Sample	SENS (%)	SPEC (%)	AUC	p
miR-21-5p	Bastaminejad 2017 [31]	80	Iran	CRC *vs* H	Serum	86.05	72.97	0.783	-
					Stool	86.05	81.08	0.829	-
				CRC I-II *vs* CRC III-IV	Stool	88.10	81.60	0.794	-
	Choi, 2019 [27]	58	South Korea	CRC *vs* NL	Stool	79.30	48.30	0.690	-
	Link, 2010 [56]	38	USA	-	Stool	-	-	-	-
	Koga, 2010 [67]	340	Japan	CRC *vs* NL	Stool	14.70	95.00	-	-
	Liu, 2016 [73]	270	China	CRC *vs* NL	Stool	90.30	75.20	0.877	-
				CRA *vs* NL		85.10	62.70	0.769	-
	Duran-Sanchon, 2020 [20]	767	Spain	AA *vs* NL	Stool	72.00	48.00	0.620	<0.05
	Liu, 2013 [83]	340	China	CRC *vs* H	Serum	65.00	85.00	0.802	-
	Jin, 2020 [61]	195	China	CRC *vs* NL	Serum	90.63	86.20	0.928	<0.05
	Yamada, 2015 [93]	188	USA	CRC *vs* NL	Serum	-	-	0.706	-
	Wang, 2014 [122]	202	China	CRC *vs* H	Serum	-	-	-	-
	Dohmen, 2022 [91]	69	Germany	CRC *vs* NL	Serum	-	-	0.550	-
	Luo, 2013 [116]	280	Germany	-	Plasma	-	-	-	-
	Bader El din, 2020 [66]	129	Egypt	CRC *vs* NL	Serum	80.70	100.00	0.936	-
	Farouk, 2020 [74]	70	Egypt	CRC *vs* H	Serum	82.86	97.15	0.893	<0.001
	Nonaka, 2015 [65]	146	Japan	CRC *vs* H	Serum	54.70	84.40	0.675	-
	Shiosaki, 2020 [72]	91	Japan	CRC *vs* NL	Serum	72.60	70.60	0.727	-
	Toiyama, 2013 [75]	279	Japan	CRC *vs* NL	Serum	82.80	90.60	0.927	-
				AA *vs* NL		76.80	81.10	0.803	-
miR-92a-3p	Chang, 2016 [29]	323	Taiwan	CRC *vs* NL	Stool	-	-	0.748	0.000
					Plasma	-	-	0.751	0.000
	Choi, 2019 [27]	58	South Korea	CRC *vs* NL	Stool	89.70	51.70	0.760	-
	Li, 2023 [84]	144	China	CRC *vs* NL	Stool	88.90	73.60	0.861	<0.001
	Liu, 2013 [83]	340	China	CRC *vs* H	Serum	65.50	82.50	0.786	-
	Huang, 2010 [82]	196	China	CRC *vs* NL	Plasma	84.00	71.20	0.838	-
				AA *vs* NL		64.60	81.40	0.749	-
	Dohmen, 2022 [91]	69	Germany	CRC *vs* NL	Serum	-	-	0.640	-
	Luo, 2013 [116]	280	Germany	-	Plasma	-	-	-	-
	Karimi, 2019 [41]	38	Iran	-	Serum	-	-	-	-
	Koga, 2010 [67]	340	Japan	CRC *vs* NL	Stool	15.70	90.80	-	-
	Shiosaki, 2020 [72]	91	Japan	-	Serum	-	-	-	-
	Wang, 2014 [122]	202	China	-	Serum	-	-	-	-
	Yamada,2015 [93]	188	USA	-	Serum	-	-	-	-
	Zheng, 2014 [104]	697	China	CRC *vs* H	Serum	-	-	0.871	-
miR-18a-5p	Chang, 2016 [29]	323	Taiwan	CRC *vs* NL	Plasma	-	-	0.715	0.000
	Zhang, 2013 [94]	164	China	CRC *vs* H	Plasma	73.10	79.10	0.804	0.004

Table S1. Cont.

MicroRNA	Author, Year	N	Country	Compared Groups	Sample	SENS (%)	SPEC (%)	AUC	p
miR-18a-5p	Herreros-Villanueva, 2019 [123]	297	Spain	-	Plasma	-	-	-	-
	Koga, 2010 [67]	340	Japan	CRC *vs* NL	Stool	47.20	94.10	-	-
	Yau, 2014 [106]	595	China	CRC *vs* NL	Stool	61.00	69.00	0.670	-
	Karimi, 2019 [41]	38	Iran	-	Serum	-	-	-	-
	Luo, 2013 [116]	280	Germany	-	Plasma	-	-	-	-
	Zhang, 2019 [95]	271	China	CRC *vs* H	Plasma	-	-	0.777	-
	Yamada, 2015 [93]	188	USA	-	Serum	-	-	-	-
miR-29a-3p	Zhu, 2016 [105]	131	China	CRC *vs* NL	Stool	85.00	61.00	0.777	<0.001
	Duran-Sanchon, 2020 [20]	767	Spain	AA *vs* NL	Stool	68.00	53.00	0.620	<0.05
	Dohmen, 2022 [91]	69	Germany	CRC *vs* NL	Serum	-	-	0.640	-
	Herreros-Villanueva, 2019 [123]	297	Spain	-	Plasma	-	-	-	-
	Luo, 2013 [116]	280	Germany	-	Plasma	-	-	-	-
	Huang, 2010 [82]	196	China	CRC *vs* NL	Plasma	69.00	89.10	0.844	-
				AA *vs* NL		73.00	79.70	0.769	-
	Shiosaki, 2020 [72]	91	Japan	-	Serum	-	-	-	-
	Yamada, 2015 [93]	188	USA	CRC *vs* NL	Serum	-	-	0.741	-
miR-20a-5p	Chang, 2016 [29]	323	Taiwan	CRC *vs* NL	Stool	-	-	0.639	0.000
	Koga, 2010 [67]	340	Japan	CRC *vs* NL	Stool	18.30	91.60	-	-
	Yau, 2015 [119]	595	China	CRC *vs* NL	Stool	55.00	82.00	0.730	-
	Luo, 2013 [116]	280	Germany	-	Plasma	-	-	-	-
	Liu, 2018 [92]	150	China	CRC *vs* H	Plasma	-	-	0.929	-
	Maminezhad, 2020 [32]	90	Iran	CRC *vs* H	Serum	-	-	0.830	<0.001
	Yamada, 2015 [93]	188	USA	-	Serum	-	-	-	-
miR-223-3p	Chang, 2016 [29]	323	Taiwan	CRC *vs* NL	Stool	-	-	0.796	0.000
					Plasma	-	-	0.707	0.000
	Phua, 2014 [44]	45	Singapore	CRC *vs* NL	Stool	76.50	96.40	0.939	
	Zhu, 2016 [105]	131	China	CRC *vs* NL	Stool	60.00	71.00	0.649	0.004
	Huang, 2010 [82]	196	China	-	Plasma	-	-	-	-
	Zheng, 2014 [104]	697	China	CRC *vs* H	Serum	-	-	0.890	-
	Karimi, 2019 [41]	38	Iran	-	Serum	-	-	-	-
miR-135b-5p	Choi, 2019 [27]	58	South Korea	-	Stool	-	-	-	-
	Phua, 2014 [44]	45	Singapore	-	Stool	-	-	-	-
	Wu, 2014 [57]	424	China	CRC *vs* NL	Stool	78.00	68.00	0.790	-
				AA *vs* NL		73.00	-	0.710	-
				CRA *vs* NL		61.00	-	-	-
				CRP *vs* NL		65.00	-	-	-
	Koga, 2010 [67]	340	Japan	CRC *vs* NL	Stool	45.70	95.00	-	-
	Karimi, 2019 [41]	38	Iran	-	Serum	-	-	-	-

Table S1. Cont.

MicroRNA	Author, Year	N	Country	Compared Groups	Sample	SENS (%)	SPEC (%)	AUC	p
miR-135b-5p	Li, 2020 [28]	106	China	CRC *vs* H	Serum	93.10	72.70	0.830	-
					Stool	96.50	74.10	0.870	-
miR-19a-3p	Koga, 2010 [67]	340	Japan	CRC *vs* NL	Stool	53.30	89.10	-	-
	Herreros-Villanueva, 2019 [123]	297	Spain	-	Plasma	-	-	-	-
	Yamada, 2015 [93]	188	USA	-	Serum	-	-	-	-
	Zheng, 2014 [104]	697	China	CRC *vs* H	Serum	-	-	0.849	-
	Dohmen, 2022 [91]	69	Germany	CRC *vs* NL	Serum	-	-	0.600	-
	Maminezhad, 2020 [32]	90	Iran	CRC *vs* H	Serum	-	-	0.870	<0.001
miR-221-3p	Chang, 2016 [29]	323	Taiwan	CRC *vs* NL	Plasma	-	-	0.743	0.000
	Yau, 2014 [106]	595	China	CRC *vs* NL	Stool	63.00	74.00	0.730	-
	Duran, 2020 [20]	767	Spain	AA *vs* NL	Stool	70.00	51.00	0.620	<0.05
	Huang, 2010 [82]	196	China	-	Plasma	-	-	-	-
	Karimi, 2019 [41]	38	Iran	-	Serum	-	-	-	-
	Shiosaki, 2020 [72]	91	Japan	-	Serum	-	-	-	-
miR-143-5p	Link, 2010 [56]	38	USA	-	Stool	-	-	-	-
	Luo, 2013 [116]	280	Germany	-	Plasma	-	-	-	-
	Huang, 2020 [69]	270	China	CRC *vs* H	Serum	-	-	0.745	<0.001
	Karimi, 2019 [41]	38	Iran	-	Serum	-	-	-	-
	Maminezhad, 2020 [32]	90	Iran	CRC *vs* H	Serum	-	-	0.760	<0.001
	Wang, 2014 [122]	202	China	-	Serum	-	-	-	-
miR-31-5p	Chang, 2016 [29]	323	Taiwan	CRC *vs* NL	Plasma	-	-	0.559	0.092
	Wu, C, 2014 [57]	424	China	-	Stool	-	-	-	-
	Karimi, 2019 [41]	38	Iran	-	Serum	-	-	-	-
	Liu, 2018 [92]	150	China	-	Plasma	-	-	-	-
	Toiyama, 2013 [75]	279	Japan	-	Serum	-	-	-	-
	Wang, 2014 [122]	202	China	-	Serum	-	-	-	-
miR-145-5p	Zhu, 2016 [105]	131	China	-	Stool	-	-	-	-
	Luo, 2013 [116]	280	Germany	-	Plasma	-	-	-	-
	Huang, 2020 [69]	270	China	CRC *vs* H	Serum	-	-	0.754	<0.001
	Maminezhad, 2020 [32]	90	Iran	CRC *vs* H	Serum	-	-	0.780	<0.001
	Wang, 2014 [122]	202	China	-	Serum	-	-	-	-
miR-27a-3p	Duran-Sanchon, 2020 [20]	767	Spain	AA *vs* NL	Stool	69.00	52.00	0.623	<0.05
	Tan, 2019 [96]	255	China	CRC *vs* H	Plasma	75.00	85.00	0.881	<0.05
	Liu, 2018 [97]	369	China	CRC *vs* H	Plasma	81.82	90.91	0.866	-
				CRC-I *vs* H		75.00	77.50	0.773	-
				CRC-I *vs* AA		80.00	77.50	0.746	-
	Farouk, 2020 [74]	70	Egypt	CRC *vs* H	Serum	42.86	88.57	0.665	0.024
miR-23a-3p	Farouk, 2020 [74]	70	Egypt	CRC *vs* H	Serum	82.86	91.34	0.887	<0.001

Table S1. Cont.

MicroRNA	Author, Year	N	Country	Compared Groups	Sample	SENS (%)	SPEC (%)	AUC	p
miR-23a-3p	Dohmen, 2022 [91]	69	Germany	CRC *vs* NL	Serum	-	-	0.670	-
	Karimi, 2019 [41]	38	Iran	CRC *vs* NL	Serum	-	-	0.890	-
	Shi, 2021 [98]	135	China	CRC-I *vs* H	Serum	89.91	74.29	0.850	-
				CRC *vs* H	Serum	91.00	74.29	0.890	-
miR-17-5p	Koga, 2010 [67]	340	Japan	CRC *vs* NL	Stool	15.70	89.10	-	-
	Link, 2010 [56]	38	USA	-	Stool	-	-	-	-
	Zhang, 2019 [95]	271	China	CRC *vs* H	Plasma	-	-	0.742	-
	Wang, 2014 [122]	202	China	-	Serum	-	-	-	-
miR-25-3p	Chang, 2016 [29]	323	Taiwan	CRC *vs* NL	Plasma	-	-	0.642	0.000
	Link, 2010 [56]	38	USA	-	Stool	-	-	-	-
	Duran-Sanchon, 2020 [20]	767	Spain	AA *vs* NL	Stool	72.00	47.00	0.620	<0.05
	Huang, 2010 [82]	196	China	-	Plasma	-	-	-	-
miR-1290	Imaoka, 2016 [45]	324	Japan	CRC *vs* H	Serum	70.10	91.20	0.830	-
				AA *vs* H		46.40	91.20	0.718	-
	Liu, 2019 [114]	205	China	CRC *vs* NL	Plasma	75.53	87.41	0.780	-
				CRA *vs* NL	Plasma	76.65	90.23	0.880	-
	Shi, 2021 [98]	135	China	CRC-I *vs* H	Serum	83.33	85.71	0.890	-
				CRC *vs* H	Serum	85.00	88.57	0.920	-
miR-146a-5p	Liu, 2016 [73]	270	China	CRC *vs* NL	Stool	77.20	68.10	0.794	-
				CRA *vs* NL		77.50	66.70	0.698	-
	Bader El din, 2020 [66]	129	Egypt	CRC *vs* NL	Serum	98.00	74.10	0.805	-
	Huang, 2010 [82]	196	China	-	Plasma	-	-	-	-
miR-34a-5p	Duran, 2020 [20]	767	Spain	AA *vs* NL	Stool	71.00	49.00	0.620	<0.05
	Wu, 2014 [118]	122	China	CRC *vs* H	Stool	76.80	93.60	-	-
	Dohmen, 2022 [91]	69	Germany	CRC *vs* NL	Serum	-	-	0.550	-
miR-24-3p	Chang, 2016 [29]	323	Taiwan	CRC *vs* NL	Plasma	-	-	0.753	0.000
	Fang, 2015 [70]	353	China	CRC *vs* H	Plasma	78.38	83.85	0.839	-
	Yamada, 2015 [93]	188	USA	-	Serum	-	-	-	-
miR-424-5p	Tan, 2019 [96]	255	China	CRC *vs* H	Plasma	79.20	93.60	0.919	<0.05
	Liu, 2018 [92]	150	China	-	Plasma	-	-	-	-
	Yamada, 2015 [93]	188	USA	-	Serum	-	-	-	-
miR-106a-5p	Choi, 2019 [27]	58	South Korea	-	Stool	-	-	-	-
	Koga, 2013 [55]	224	Japan	CRC *vs* NL	Stool	34.20	97.20	-	-
	Link, 2010 [56]	38	USA	-	Stool	-	-	-	-
miR-106b-5p	Chang, 2016 [29]	323	Taiwan	CRC *vs* NL	Stool	-	-	0.705	0.000
	Huang, 2020 [69]	270	China	CRC *vs* H	Serum	-	-	0.624	<0.001
	Luo, 2013 [116]	280	Germany	-	Plasma	-	-	-	-
miR-200c-3p	Choi, 2019 [27]	58	South Korea	-	Stool	-	-	-	-
	Zhang, 2013 [94]	164	China	CRC *vs* NL	Plasma	64.10	73.30	0.749	0.021

Table S1. Cont.

MicroRNA	Author, Year	N	Country	Compared Groups	Sample	SENS (%)	SPEC (%)	AUC	p
miR-200c-3p	Huang, 2020 [69]	270	China	CRC *vs* H	Serum	-	-	0.656	<0.05
miR-19b-3p	Koga, 10 [67]	340	Japan	CRC *vs* NL	Stool	15.70	90.80	-	-
miR-19b-3p	Herreros-Villanueva, 2019 [123]	297	Spain	-	Plasma	-	-	-	-
miR-19b-3p	Yamada, 2015 [93]	188	USA	-	Serum	-	-	-	-
miR-16-5p	Chang, 2016 [29]	323	Taiwan	CRC *vs* NL	Stool	-	-	0.703	0.000
miR-16-5p	Wang, 2014 [122]	202	China	-	Serum	-	-	-	-
miR-16-5p	Dohmen, 2022 [91]	69	Germany	CRC *vs* NL	Serum	-	-	0.670	-
miR-181b-5p	Huang, 2010 [82]	196	China	-	Plasma	-	-	-	-
miR-181b-5p	Wang, 2014 [122]	202	China	-	Serum	-	-	-	-
miR-181b-5p	Luo, 2013 [116]	280	Germany	-	Plasma	-	-	-	-
miR-144-5p	Choi, 2019 [27]	58	South Korea	CRC *vs* NL	Stool	78.90	66.70	0.770	-
miR-144-5p	Kalimutho, 2011 [30]	75	Italy	CRC *vs* NL	Stool	74.29	87.20	0.829	-
miR-let-7c	Bader El din, 2020 [66]	129	Egypt	CRC *vs* NL	Serum	77.60	96.20	0.855	-
miR-let-7c	Dohmen, 2022 [91]	69	Germany	CRC *vs* NL	Serum	-	-	0.640	-
miR-103a-3p	Nonaka, 2015 [65]	146	Japan	CRC *vs* H	Serum	75.00	55.90	0.662	-
miR-103a-3p	Zhang, 2019 [95]	271	China	CRC *vs* H	Plasma	-	-	0.756	-
miR-601	Wang, 2012 [115]	191	China	CRC *vs* H	Plasma	62.20	72.40	0.747	0.002
miR-601	Wang, 2012 [115]	191	China	AA *vs* H	Plasma	72.10	51.70	0.638	0.0174
miR-601	Yamada, 2015 [93]	188	USA	-	Serum	-	-	-	-
miR-760	Wang, 2012 [115]	191	China	CRC *vs* H	Plasma	80.00	72.40	0.788	<0.001
miR-760	Wang, 2012 [115]	191	China	AA *vs* H		69.80	62.10	0.682	0.0214
miR-760	Yamada, 2015 [93]	188	USA	-	Serum	-	-	-	-
miR-592	Liu, 2018 [92]	150	China	-	Plasma	-	-	-	-
miR-592	Pan, 2020 [34]	246	China	CRC *vs* NL	Serum	82.80	78.00	0.844	<0.001
miR-592	Pan, 2020 [34]	246	China	CRC-I *vs* NL	Serum	78.60	80.00	0.801	<0.001
miR-592	Pan, 2020 [34]	246	China	AA *vs* NL	Serum	68.60	78.10	0.747	<0.001
miR-451	Phua, 2014 [44]	45	Singapore	CRC *vs* NL	Stool	88.20	100.00	0.971	-
miR-451	Dohmen, 2022 [91]	69	Germany	CRC *vs* NL	Serum	-	-	0.650	-
miR-335-3p	Duran-Sanchon, 2020 [20]	767	Spain	AA *vs* NL	Stool	74.00	45.00	0.620	<0.05
miR-335-3p	Herreros-Villanueva, 2019 [123]	297	Spain	-	Plasma	-	-	-	-
miR-622	Link, 2010 [56]	38	USA	-	Stool	-	-	-	-
miR-622	Ng, 2017 [68]	207	China	CRC *vs* NL	Serum	87.80	63.50	-	-
miR-18b-5p	Karimi, 2019 [41]	38	Iran	-	Serum	-	-	-	-
miR-18b-5p	Zhang, 2019 [95]	271	China	CRC *vs* H	Plasma	-	-	0.781	-
miR-320a-3p	Huang, 2010 [82]	196	China	-	Plasma	-	-	-	-
miR-320a-3p	Fang, 2015 [70]	353	China	CRC *vs* H	Plasma	92.74	73.08	0.886	-
miR-191-5p	Chang, 2016 [29]	323	Taiwan	CRC *vs* NL	Plasma	-	-	0.714	0.000
miR-191-5p	Huang, 2010 [82]	196	China	-	Plasma	-	-	-	-
miR-203a-5p	Wang, 2014 [122]	202	China	-	Serum	-	-	-	-

Table S1. Cont.

MicroRNA	Author, Year	N	Country	Compared Groups	Sample	SENS (%)	SPEC (%)	AUC	p
miR-203a-5p	Huang, 2020 [69]	270	China	CRC *vs* H	Serum	-	-	0.713	<0.001
miR-17-3p	Choi, 2019 [27]	58	South Korea	CRC *vs* NL	Stool	70.80	67.90	0.710	-
	Huang, 2010 [82]	196	China	-	Plasma	-	-	-	-
miR-135a-5p	Choi, 2019 [27]	58	South Korea	-	Stool	-	-	-	-
	Koga, 2010 [67]	340	Japan	CRC *vs* H	Stool	15.20	100.00	-	-
miR-378a-3p	Kalimutho, 2011 [30]	75	Italy	-	Stool	-	-	-	-
	Liu, 2018 [92]	150	China	-	Plasma	-	-	-	-
miR-222-3p	Huang, 2010 [82]	196	China	-	Plasma	-	-	-	-
	Dohmen, 2022 [91]	69	Germany	CRC *vs* NL	Serum	-	-	0.630	-
miR-133a-5p	Liu, 2018 [92]	150	China	-	Plasma	-	-	-	-
	Luo, 2013 [116]	280	Germany	-	Plasma	-	-	-	-
miR-532-3p	Luo, 2013 [116]	280	Germany	-	Plasma	-	-	-	-
	Kalimutho, 2011 [30]	75	Italy	-	Stool	-	-	-	-
miR-139-3p	Ng, 2017 [68]	207	China	CRC *vs* NL	Serum	96.60	97.80	0.994	-
miR-4516	Jin, 2020 [61]	195	China	CRC *vs* NL	Serum	94.40	89.80	0.958	<0.05
miR-144-3p	Tan, 2019 [96]	255	China	CRC *vs* H	Plasma	93.80	78.70	0.887	<0.05
miR-199a-5p	Zhao, 2022 [113]	233	China	CRC *vs* H	Serum	93.00	70.00	0.900	<0.001
				AA *vs* H		90.00	53.00	0.760	<0.001
				CRC *vs* AA		81.00	70.00	0.830	<0.001
miR-423-5p	Fang, 2015 [70]	353	China	CRC *vs* H	Plasma	91.89	70.77	0.833	-
miR-940	Shi, 2021 [98]	135	China	CRC-I *vs* H	Serum	90.00	71.43	0.830	-
				CRC *vs* H	Serum	90.00	77.14	0.880	-
miR-320d	Liu, 2019 [114]	205	China	CRC *vs* NL	Plasma	88.83	71.66	0.740	-
				CRA *vs* NL	Plasma	79.64	71.55	0.810	-
miR-196b	Xu, 2017 [21]	254	China	CRC *vs* H	Serum	87.38	63.00	0.814	-
miR-627-5p	Zhao, 2022 [113]	233	China	CRC *vs* H	Serum	87.00	100,00	0.970	<0.001
				AA *vs* H		62.00	93.00	0.840	<0.001
				CRC *vs* AA		71.00	93.00	0.900	<0.001
miR-126-3p	Shi, 2021 [98]	135	China	CRC *vs* H	Serum	84.00	88.57	0.900	-
				CRC-I *vs* H	Serum	86.67	77.14	0.940	-
miR-425-5p	Tan, 2019 [96]	255	China	CRC *vs* H	Plasma	83.30	91.50	0.910	<0.05
miR-130b-3p	Duran-Sanchon, 2020 [20]	767	Spain	AA *vs* NL	Stool	82.00	39.00	0.640	<0.05
miR-1260b	Tan, 2019 [96]	255	China	CRC *vs* H	Plasma	81.30	83.30	0.848	<0.05
miR-421	Duran-Sanchon, 2020 [20]	767	Spain	AA *vs* NL	Stool	81.00	43.00	0.680	<0.05
miR-148a-3p	Tan, 2019 [96]	255	China	CRC *vs* H	Plasma	79.20	91.50	0.871	<0.05
miR-182-5p	Liu, 2018 [92]	150	China	CRC *vs* NL	Plasma	78.00	91.00	0.801	-
miR-26a-5p	Bader El din, 2020 [66]	129	Egypt	CRC *vs* NL	Serum	77.60	96.20	0.918	-
miR-506-3p	Krawczyk, 2017 [64]	126	Poland	CRC *vs* H	Plasma	76.80	60.70	0.747	-
miR-338-5p	Bilegsaikhan, 2018 [63]	210	China	CRC *vs* NL	Serum	76.30	92.50	0.923	-

Table S1. Cont.

MicroRNA	Author, Year	N	Country	Compared Groups	Sample	SENS (%)	SPEC (%)	AUC	p
miR-224-5p	Zhu, 2016 [105]	131	China	CRC *vs* NL	Stool	75.00	63.00	0.774	<0.001
miR-143-3p	Tan, 2019 [96]	255	China	CRC *vs* H	Plasma	72.90	78.70	0.777	<0.05
miR-130a-3p	Liu, 2018 [97]	369	China	CRC *vs* H	Plasma	69.32	100.00	0.816	-
				CRC-I *vs* H		82.50	62.50	0.742	-
				CRC *vs* AA		70.00	80.00	0.697	-
miR-4316	Krawczyk, 2017 [64]	126	Poland	CRC *vs* H	Plasma	60.90	83.90	0.744	-
miR-720	Nonaka, 2015 [65]	146	Japan	CRC *vs* H	Serum	58.30	56.30	0.630	-
miR-422a	Zheng, 2014 [104]	697	China	CRC *vs* H	Serum	-	-	0.843	-
miR-301a-3p	Karimi, 2019 [41]	38	Iran	CRC *vs* NL	Serum	-	-	0.840	-
miR-125b-5p	Yamada, 2015 [93]	188	USA	CRC *vs* NL	Serum	-	-	0.806	-
miR-150-5p	Maminezhad, 2020 [32]	90	Iran	CRC *vs* H	Serum	-	-	0.750	<0.001
miR-151a-5p	Zhang, 2019 [95]	271	China	CRC *vs* H	Plasma	-	-	0.737	-
miR-181a-5p	Zhang, 2019 [95]	271	China	CRC *vs* H	Plasma	-	-	0.736	-
miR-127-3p	Zhang, 2019 [95]	271	China	CRC *vs* H	Plasma	-	-	0.729	-
miR-375-3p	Huang, 2020 [69]	270	China	CRC *vs* H	Serum	-	-	0.715	<0.001
miR-let-7a-3p	Maminezhad, 2020 [32]	90	Iran	CRC *vs* H	Serum	-	-	0.710	0.0048
miR-200c-5p	Huang, 2020 [69]	270	China	CRC *vs* H	Serum	-	-	0.702	<0.001
miR-10b	Chang, 2016 [29]	323	Taiwan	CRC *vs* NL	Plasma	-	-	0.645	0.000
miR-141-3p	Chang, 2016 [29]	323	Taiwan	CRC *vs* NL	Plasma	-	-	0.581	0.021
miR-497-5p	Chang, 2016 [29]	323	Taiwan	CRC *vs* NL	Plasma	-	-	0.576	0.031
miR-145-3p	Huang, 2020 [69]	270	China	CRC *vs* H	Serum	-	-	0.525	<0.05
miR-29b-3p	Link, 2010 [56]	38	USA	-	Stool	-	-	-	-
miR-654-3p	Link, 2010 [56]	38	USA	-	Stool	-	-	-	-
miR-1238-3p	Link, 2010 [56]	38	USA	-	Stool	-	-	-	-
miR-938	Link, 2010 [56]	38	USA	-	Stool	-	-	-	-
miR-576-3p	Eldaly, 2020 [22]	28	Egypt	-	Serum	-	-	-	-
miR-613	Eldaly, 2020 [22]	28	Egypt	-	Serum	-	-	-	-
miR-134-5p	Huang, 2010 [82]	196	China	-	Plasma	-	-	-	-
miR-142	Karimi, 2019 [41]	38	Iran	-	Serum	-	-	-	-
miR-675-5p	Liu, 2018 [92]	150	China	-	Plasma	-	-	-	-
miR-875-3p	Liu, 2019 [114]	205	China	-	Plasma	-	-	-	-
miR-490-5p	Liu, 2019 [114]	205	China	-	Plasma	-	-	-	-
miR-1266	Liu, 2019 [114]	205	China	-	Plasma	-	-	-	-
miR-615-3p	Liu, 2019 [114]	205	China	-	Plasma	-	-	-	-
miR-let-7g-5p	Wang, 2014 [122]	202	China	-	Serum	-	-	-	-
miR-30c-5p	Wang, 2014 [122]	202	China	-	Serum	-	-	-	-
miR-409-3p	Yamada, 2015 [93]	188	USA	-	Serum	-	-	-	-
miR-575	Yamada, 2015 [93]	188	USA	-	Serum	-	-	-	-
mir-342-3p	Luo, 2013 [116]	280	Germany	-	Plasma	-	-	-	-

Table S1. Cont.

MicroRNA	Author, Year	N	Country	Compared Groups	Sample	SENS (%)	SPEC (%)	AUC	p
miR-15b-5p	Herreros-Villanueva, 2019 [123]	297	Spain	-	Plasma	-	-	-	-

Abbreviation: miR: microRNA; NL: No Lesion; CRP: Colorectal polyps; CRA: Colorectal Adenoma; AA: Advanced Adenoma; CRC: Colorectal Cancer; CRC-I: CRC in stage I; CRC-II: CRC in stage II; CRC-III: CRC in stage III; CRC-IV: CRC in stage IV; CRC-I-II: CRC in stage I or II; CRC-III-IV: CRC in stage III or IV; AUC: Area under the curve; p: p asymptotic.

Table S2. Performance of microRNAs combinations described in the context CRC screening both in stool and blood samples.

MicroRNAs combinations	Author, year	N	Country	Compared Groups	Sample	SEN/SPE (%)	AUC	p
miR-223-3p + miR-92a-3p	Chang, 2016 [29]	323	Taiwan	CRC *vs* NL	Stool and Plasma	96.80/ 75.00	0.910	-
miR-21-5p + miR-23a-3p + miR-27a-3p	Farouk, 2020 [74]	70	Egypt	CRC *vs* H	Serum	97.14/ 82.86	0.908	<0.001
miR-21-5p + miR-23a-3p	Farouk, 2020 [74]	70	Egypt	CRC *vs* H	Serum	97.14/ 82.86	0.905	<0.001
miR-144-3p + miR-425-5p + miR-1260b	Tan, 2019 [96]	255	China	CRC *vs* H	Plasma	93.80/ 91.30	0.954	<0.05
miR-331-3p + miR-15b-5p + miR-25-5p	Kanaan, 2013 [62]	128	USA	CRC *vs* NL	Plasma	93.00/ 74.00	-	-
miR-4516 + miR-21-5p	Jin, 2020 [61]	195	China	CRC *vs* NL	Serum	92.11/ 87.60	0.943	<0.001
miR-21-5p + miR-26a-5p	Bader El Din, 2020 [66]	129	Egypt	CRC *vs* NL	Serum	91.80/ 91,.70	-	-
miR-331-3p + miR-15b-5p + miR-21-5p + miR-142-3p + miR-339-3p	Kanaan, 2013 [62]	128	USA	CRC *vs* AA	Plasma	91.00/ 69.00	0.856	-
miR-431-5p + miR-139-3p	Kanaan, 2013 [62]	128	USA	CRC *vs* NL	Plasma	91.00/ 57.00	0.829	-
miR-19a-3p + miR-19b-3p + miR-15b-5p + miR-29a-3p + miR-335-3p + miR-18a-5p	Herreros-Villanueva, 2019 [123]	297	Spain	CRC *vs* NL	Plasma	91.00/ 90.00	0.949	-
				AA *vs* H		95.00/ 90.00	0.914	-
				(CRC; AA) vs H		85.00/ 90.00	0.916	-
miR-513a-5p + miR-628-3p + miR-193a-5p + miR-210-3p	Nakamura, 2022 [121]	349	Barcelona	CRC-I *vs* H	Plasma	90.00/ 80.00	0.920	<0.001
				CRC-I *vs* CRC-II		92.00/ 80.00	0.920	<0.001
				CRC-III *vs* CRC-IV		79.00/ 86.00	0.870	<0.001
				CRC III-IV *vs* H		82.00/ 88.00	0.840	<0.001
miR-126-3p + miR-1290 + miR-23a-3p + miR-940	Shi, 2021 [98]	135	China	CRC-I *vs* H	Serum	90.00/ 82.86	0.920	-
miR-126-3p + miR-1290 + miR-23a-3p + miR-940	Shi, 2021 [98]	135	China	CRC *vs* H	Serum	90.00/ 88.57	0.950	-
miR-532-3p + miR-331-3p + miR-195-5p + miR-17-5p + miR-142-3p + miR-15b-5p + miR-532-3p + miR-652-3p	Kanaan, 2013 [62]	128	USA	CRC *vs* NL	Plasma	88.00/ 64.00	0.868	-
miR-627-5p + miR-199a-5p	Zhao, 2022 [113]	233	China	CRC *vs* H	Serum	87.10/ 100.00	0.980	<0.001
				AA *vs* H		78.00/ 70.00	0.860	<0.001
				CRC *vs* AA		83.00/ 87.00	0.920	<0.001
miR-21-5p + miR-27a-3p	Farouk, 2020 [74]	70	Egypt	CRC *vs* H	Serum	85.71/ 88.57	0.899	<0.001
miR-27a-3p + miR-130a-3p	Liu, 2018 [97]	369	China	CRC *vs* H	Plasma	85.23/ 90.91	0.899	-
				CRC-I *vs* H	Plasma	82.50/ 75.00	0.846	-
				AA vs H	Plasma	80.00/ 90.00	0.801	-
miR-200c-5p + miR-18a-5p	Zhang, 2013 [104]	164	China	CRC *vs* NL	Plasma	84.60/ 75.60	0.839	-
let-7c + miR-146a-5p	Bader El Din, 2020 [66]	129	Egypt	CRC *vs* NL	Serum	84.50/ 88.00	-	-

Table S2. Cont.

MicroRNAs combinations	Author, year	N	Country	Compared Groups	Sample	SEN/SPE (%)	AUC	p
miR-92a-3p + miR-223-3p + miR-19a-3p + miR-422a	Zheng, 2014 [104]	697	China	CRC *vs* H	Serum	84.30/ 91.60	0.951	<0.001
				CRC *vs* CRA		-/-	0.886	-
				CRA *vs* H		-/-	0.705	-
miR-21-5p + let-7g-5p + miR-92a-3p + miR-181b-5p + miR-31-5p + miR-203a-3p	Wang, 2014 [122]	202	China	CRC *vs* H	Serum	83.30/ 96.70	0.923	-
miR-29a-5p + miR-92a-3p + miR-760	Wang, 2012 [115]	191	China	CRC *vs* H	Plasma	83.30/ 93.10	0.943	-
miR-601 + miR-760	Wang, 2012 [115]	191	China	CRC *vs* H	Plasma	83.30/ 69.10	0.792	-
				AA *vs* H	Plasma	72.10/ 62.10	0.683	-
let-7c + miR-146a-5p + miR-21-5p + miR-26a-5p	Bader El Din, 2020 [66]	129	Egypt	CRC *vs* NL	Serum	82.10/ 100.00	0.953	0.001
miR-203a-3p + miR-145-5p + miR-375-3p + miR-200c-5p	Huang, 2020 [69]	270	China	CRC *vs* H	Serum	81.25/73.33	0.893	-
miR-1290 + miR-320d	Liu, 2019 [114]	150	China	CRC *vs* NL	Plasma	81.21/ 90.74	0.910	-
				AA *vs* NL	Plasma	77.46/ 88.69	0.820	-
miR-18a-5p + miR-92a-3p + miR-221-3p + miR-223-3p + miR-191-5p + miR-24-3p	Chang, 2016 [29]	323	Taiwan	CRC *vs* NL	Plasma	76.30/ 68.80	0.790	-
miR-223-3p + miR-92a-3p	Chang, 2016 [29]	323	Taiwan	CRC *vs* NL	Plasma	75.80/ 70.50	0.780	-
miR-92a-3p + miR-29a-3p	Huang, 2010 [82]	196	China	CRC *vs* NL	Plasma	83.00/ 84.70	0.883	<0.001
				AA *vs* NL		73.00/ 79.70	0.773	<0.001
miR-24 + miR-320a + miR-423-5p	Fang, 2015 [70]	353	China	CRC *vs* H	Plasma	70.77/ 92.79	0.899	-
miR-21-5p + miR-92a-3p	Liu, 2013 [83]	340	China	CRC *vs* H	Serum	68.00/91.20	0.847	-
				CRC *vs* AA		70.00/70.00	0.722	-
miR-654-5p + miR-126-3p + miR-10b + miR-144-3p	Du, 2022 [120]	220	China	CRC *vs* H	Serum	-/-	0.913	<0.05
miR-20a-5p + miR-182-5p	Liu, 2018 [92]	150	China	CRC *vs* NL	Serum	-/-	0.905	-
let-7c + miR-16-5p + miR-21-5p + miR-23a-3p + miR-29a-3p + miR-34a-5p + miR-92a-3p + miR-222-3p + miR-451	Dohmen, 2022 [91]	69	Germany	CRC *vs* NL	Serum	-/-	0.900	0.05
miR-21-5p + miR-29a-3p + miR-125b-5p	Yamada, 2015 [93]	188	USA	CRC *vs* NL	Serum	-/-	0.828	-
miR-18a-5p + miR-20a-5p + miR-21-5p + miR-92a-3p + miR-143-5p + miR-145-5p + miR-181b-5p + miR-29a-3p + miR-106b-5p + miR-133a-5p + miR-342-3p + miR-532-3p	Lou, 2013 [116]	280	Germany	CRC + H	Plasma	-/-	0.803	-
miR-16-5p + miR-19a-3p + miR-21-5p + miR-34a-5p + miR-222-3p	Dohmen, 2022 [91]	69	Germany	CRC *vs* NL	Serum	-/-	0.800	0.05
miR-16-5p + miR-34a-5p	Dohmen, 2022 [91]	69	Germany	CRC *vs* NL	Serum	-/-	0.740	0.05

Table S2. Cont.

MicroRNAs combinations	Author, year	N	Country	Compared Groups	Sample	SEN/SPE (%)	AUC	p
miR-21-5p + miR-29a-3p + miR-92a-3p	Shiosaki, 2020 [72]	91	Japan	CRC *vs* NL	Serum	-/ -	0.639	-
miR-19a-5p + miR-20a-5p + miR-143-5p + miR-145-5p + miR-150-5p + let-7a-3p	Maminezhad, 2020 [32]	90	Iran	-	Serum	-/ -	-	-
miR-103a-3p + miR-127-3p + miR-151a-5p + miR-17-5p + miR-181a-5p + miR-18a-5p + miR-18b-5p	Zhang, 2019 [95]	271	China	-	Plasma	-/ -	-	-
miR-34b-3p + miR-34c-5p	Wu, 2014 [118]	122	China	CRC *vs* H	Stool	95.00/ 100.00	-	-
miR-92a-3p + miR-144-5p	Choi, 2019 [27]	58	South Korea	CRC *vs* NL	Stool	96.60/ 37.90	-	-
miR-21-5p + miR-146a-5p	Liu, 2016 [73]	270	China	CRC *vs* H	Stool	87.00/ 81.70	0.878	-
				CRA *vs* H		78.90/ 66.80	0.761	-
miR-20a-5p + miR-135b-5p	Yau, 2015 [119]	595	China	CRC *vs* NL	Stool	79.00/ 65.00	0.790	-
miR-223-3p + miR-92a-3p + miR-16-5p + miR-106b-5p	Chang, 2016 [29]	323	Taiwan	CRC *vs* NL	Stool	73.90/ 82.20	0.840	-
miR-223-3p + miR-92a-3p	Chang, 2016 [29]	323	Taiwan	CRC *vs* NL	Stool	71.70/ 79.90	0.810	-
miR-18a-5p + miR-221-3p	Yau 2014 [106]	595	China	CRC *vs* NL	Stool	66.00/ 75.00	0.750	-
miR-135a-5p + miR-135b-5p	Wu, 2014 [57]	424	China	CRA *vs* NL	Stool	61.00/ 58.40	-	-
				AA *vs* NL		73.00/ 58.80	-	-
				CRC-I-II *vs* NL		67.00/ 58.40	-	-
				CRC-III-IV *vs* NL		80.00/ 58.40	-	-
miR-20a-5p + miR-92a-3p	Yau, 2015 [119]	595	China	CRC *vs* NL	Stool	57.00/ 84.00	0.770	-
miR-27a-3p + miR-421	Duran-Sanchon, 2020 [20]	767	Spain	AA *vs* NL	Stool	59.00/-	0.930	-

Abbreviation: miR: microRNA; NL: No Lesion; CRA: Colorectal Adenoma; AA: Advanced Adenoma; CRC: Colorectal Cancer; CRC-I: CRC in stage I; CRC-II: CRC in stage II; CRC-III: CRC in stage III; CRC-IV: CRC in stage IV; CRC-I-II: CRC in stage I or II; CRC-III-IV: CRC in stage III or IV; AUC: Area under the curve; p: p asymptotic.
